# Justifying the need for a recovery related surveillance system: Exploratory focused interviews

**DOI:** 10.1002/hsr2.2038

**Published:** 2024-04-21

**Authors:** Joseph Ellis, Grace L. Clancy, Amber Kizewski, Tyler Jennings, Robin A. Thompson, Paula Arnett, Terry L. Bunn

**Affiliations:** ^1^ Kentucky Injury Prevention and Research Center University of Kentucky College of Public Health Lexington Kentucky USA; ^2^ Fletcher Group, Inc. London Kentucky USA; ^3^ Substance Use Priority Research Area University of Kentucky Lexington Kentucky USA; ^4^ Get Help Tarzana California USA; ^5^ University of Kentucky College of Public Health Lexington Kentucky USA; ^6^ Department of Epidemiology and Environmental Health University of Kentucky College of Public Health Lexington Kentucky USA

**Keywords:** data collection, recovery housing, substance use disorder, surveillance system

## Abstract

**Background and Aims:**

No recovery related surveillance system exists but given the evidence of effectiveness and growing supply, a house‐ and resident‐ level recovery house (RH) surveillance system could be beneficial for data collection on recovery support service (RSS) engagement, and retention; for improved standardization of RH programs and services; and for identification of outcomes associated with long‐term recovery.

**Methods:**

This study aimed to explore current data collection practices at the resident‐ and house‐ level through qualitative focus interviews of RH representatives. The 13 RH interviews were scheduled with 16 RH representative respondents.

**Results:**

The most frequently collected resident data was at entry (92%) and departure (85%) and included demographics (*n* = 5), substance use history (*n* = 6), treatment and recovery history (*n* = 5), legal and correctional history (*n* = 6) and mental health information (*n* = 7). Recovery support data was collected by 85% of houses. Post‐stay data was only collected by four RHs (31%).

**Conclusion:**

These results indicate that there is a lack of standardized systematic collection, analysis, and reporting of recovery related data in the RH field. A recovery related surveillance system has the potential to fill this gap and inform and improve standard of resident care to support long‐term recovery from substance use disorder.

## 1 INTRODUCTION

Substance use disorder (SUD) has widespread societal impacts costing the United States ~$3.7 trillion in 2019, spanning costs related to productivity loss, healthcare, criminal justice, substance use related crashes, and public assistance and social services.^1^ SUD treatment can help individuals initiate long‐term recovery, and recovery from SUD requires a multipronged approach ensuring that health related social needs are met. Recovery housing (RH) is defined by the Substance Abuse and Mental Health Services Administration (SAMHSA) as “safe, healthy, family‐like substance free living environments that support individuals in recovery from addiction. While recovery residences vary widely in structure, all are centered on peer support connection to services that promote long‐term recovery.^2^” RH can reduce substance use, increase incomes, and decrease recidivism.^3^, ^4^, ^5^ The exact number of recovery houses nationwide is unknown; estimates indicate there are 10,358–17,900 nationwide.^6^, ^7^

Public health surveillance is defined as “ongoing systematic collection, analysis, and interpretation of health‐related data essential to planning, implementation, and evaluation of public health practice.”^8^ Currently, no recovery related surveillance system exists but given the evidence of effectiveness and growing supply, a house‐ and resident‐ level RH surveillance system could be beneficial for data collection on recovery support service (RSS) engagement, and retention; for improved standardization of RH programs and services; and for identification of outcomes associated with long‐term recovery. This study aims to explore current data collection practices at the resident‐ and house‐ level through qualitative focus interviews of RH representatives.

## 2 METHODS

### 2.1 Interview guide development

A semi‐structured interview guide was designed and reviewed by subject matter experts to gather comprehensive information about RH resident and house data collection, data reporting, and processes/procedures. The study was approved by the University of Kentucky IRB #53931.

### 2.2 Participant recruitment

A purposive sampling approach was employed; potential candidates were identified through the Rural Center of Excellence in Recovery. A recruitment email outlining the study was sent and candidates responsive were sent an invitation for a 1‐h interview. Candidates unresponsive to the initial invitation were sent a reminder email after 2 weeks, and ~4 weeks. Data collection occurred in December 2020.

### 2.3 Interview process

The 13 RH interviews were scheduled with 16 RH representative respondents and were randomly assigned to study team members. The RH respondents included RH executives, owners, managers, and other RH administrators. Consent was obtained before the interview. The meetings were recorded in, and automatically transcribed by Zoom.

### 2.4 Code book development

A hybrid coding approach of inductive and deductive processes was employed.^9^ The deductive approach resulted in three categories of codes identified (Data Collection and Reporting, RH Processes and Procedures, and Website Recommendations) based on the interview questions. After codebook development, an inductive method was employed for codebook usability testing. The study team coded two transcripts each, searching for code utility and gaps. When code gaps were found, new codes were developed with group consensus.

### 2.5 Interview data analysis

Intercoder reliability scores for were 0.87, calculated utilizing percent agreement between two interviews chosen at random and averaging scores. Interview coding was conducted in two rounds. In each round, two study team members independently coded each transcript in Microsoft Word then Excel was used to compile interview code data.^10^ The first round of coding also tested the codebook to ensure codes were comprehensive. No codebook edits were required after round one was completed. In round two, two study team members coded the same transcript independently. Once coding rounds were completed, an additional study team member input all codes into one Excel document and checked for coding discrepancies. Coding discrepancies were resolved by study team members.

All coded data was analyzed in Excel, and qualitative themes were identified. Some themes were re‐organized to create subthemes.^11^ Identified themes included Mental Health, Recovery History, Medications, Manual Data Collection, and External Recipients of Reports.

## 3 RESULTS

The 13 RHs participated; 38% were located in rural areas (based on Health Resources and Services Administration (HRSA) definition) and 62% were located in urban areas (HRSA, 2022). The 31% were located in Oregon, 15% in Montana, 15% in West Virginia, 15% in Kentucky, 8% in Idaho, 8% in Ohio, and 8% in Washington.

The most frequently collected resident data was at entry (92%) and departure (85%) and included demographics (*n* = 5), substance use history (*n* = 6), treatment and recovery history (*n* = 5), legal and correctional history (*n* = 6) and mental health information (*n* = 7) (Appendix 1). Recovery support data was collected by 85% of houses. Post‐stay data was only collected by four RHs (31%).

Forty‐six percent indicated that resident progress was important to track; and 46% (*n* = 6) mentioned the importance of tracking recovery goals and RSS like social support, external program involvement, and job status. The majority (85%) collected RH program involvement and progress data; 73% collected this data digitally. One respondent that didn't collect data digitally stated:

*“I would love to have a technology piece to do that for me, instead of carrying around my shrewd notebook.”*

The 11 houses (85%) collected RH financial information (Appendix 2); 77% (*n* = 10) collected data on resident rent. One respondent described using a pre‐existing software system for tracking financial data.

Sixty‐two percent reported RH data to external organizations; 54% reported to a public funder. Four RHs indicated having a system that could produce business related reports related to RH management would be beneficial to understand financials. For example, one respondent stated:

*“From a business perspective… the other metric that I think is important, that we track, but I think is just important in general is figuring out what your optimal revenue per month would be for the home. So, how much money could you feasibly take in if everyone paid in full on time versus the actual revenue that you're generating… if you're losing money left and right at a certain point, you [have] to close down.”*

Eleven RHs reported that having a system that could produce resident‐level reports would be beneficial. Forty‐six percent tracked room and bed availability digitally and 54% collected availability data manually.

## 4 DISCUSSION

Results from this exploratory qualitative study with 13 RHs across seven states indicated that limited recovery resident data is regularly collected. Regular collection and analysis of resident background information, SUD history, such as medication use, prior substance use and severity of use, and recovery capital has the potential to enhance in‐house and local community RSS available to RH residents.^12^ Also, few RHs collected social support and other RSS data that are critical in assessing individuals' progress in initiating recovery.^12^ Additionally, regular collection and analysis of resident data has the potential to identify disparities by race, gender, and geographic location,^13^ and to inform development of tailored individualized RH programs and interventions. While most RHs collected resident entry and exit data, follow‐up outcome data was rarely collected. A recovery related surveillance system with intake, departure, and long‐term resident outcome data could examine RSSs associated with long‐term SUD recovery; inform and improve RSS interventions; support outcomes informed recovery care; allow cost comparability of RH nationally; and to improve RH program quality and associated resident outcomes.

This study has limitations; it had a small sample size of 13 RHs, thus RH data provided by respondents may not be representative of the national landscape of RH data collection practices. There could be selection bias since this study did not include RHs without email capabilities. It is also possible that only interested RHs responded to the interview request.

## 5 CONCLUSIONS

There is a lack of standardized systematic collection, analysis, and reporting of recovery related data in the RH field. A recovery related surveillance system has the potential to fill this gap and inform and improve standard of resident care to support long‐term recovery from SUD.

## AUTHOR CONTRIBUTIONS

**Joseph Ellis**: Conceptualization; data curation; formal analysis; investigation; methodology; writing—original draft. **Grace L. Clancy**: Conceptualization; data curation; formal analysis; investigation; methodology; writing—original draft. **Amber Kizewski**: Formal analysis; investigation; methodology; resources. **Tyler Jennings**: Data curation; investigation. **Robin A. Thompson**: Data curation; formal analysis; investigation; methodology. **Paula Arnett**: Investigation; methodology. **Terry L. Bunn**: Conceptualization; formal analysis; funding acquisition; methodology; writing—original draft; writing—review and editing.

## CONFLICT OF INTEREST STATEMENT

No conflicts declared.

## TRANSPARENCY STATEMENT

The lead author Terry L. Bunn affirms that this manuscript is an honest, accurate, and transparent account of the study being reported; that no important aspects of the study have been omitted; and that any discrepancies from the study as planned (and, if relevant, registered) have been explained.

## Data Availability

Deidentified interview data is available to researchers upon reasonable request with a data use and management plan and IRB approval.

## APPENDIX 1

Table A1

**Table A1:** Resident level data collection (*N* = 13), United States, 2020.

Question	Recovery house responses
**1. What information is typically collected about residents during their stay in a recovery house?**	
Types of data collection	
Entry data collection	*n* = 12 (92%)
Departure data collection	*n* = 11 (85%)
Poststay data collection	*n* = 4 (31%)
Demographics	
Age	*n* = 5 (38%)
Race/ethnicity	*n* = 5 (38%)
Education level	*n* = 5 (38%)
Substance use history	
Medications	*n* = 8 (62%)
Drug use history	*n* = 6 (46%)
Legal and correctional history	*n* = 6 (46%)
Mental health	*n* = 7 (54%)
Treatment and recovery history	
Treatment history	*n* = 5 (38%)
Sober date/recovery history	*n* = 5 (38%)
Recovery supports	*n* = 11 (85%)
Urine screen	*n* = 4 (31%)
**2. In terms of recovery capital (such as social support and 12‐step affiliation), recovery ecosystem (workforce training, employment, etc.), and recovery outcomes (sustained recovery, improved quality of life) ‐ in your opinion, what type of information is important to track and record?**	
Success/progress	*n* = 6 (46%)
Recovery supports	
Social supports (peer, family, etc.)	*n* = 6 (46%)
External program involvement (12‐Step, AA, NA, Food support, etc.)	*n* = 6 (46%)
Job status	*n* = 6 (46%)
Recovery goals	*n* = 6 (46%)
**3. Do you document program involvement and progress for your residents during their stay? If so, please describe how you keep track of this information. If not, would you like to have an easy system to document this information?**	
**Yes ‐ collecting program involvement data**	*n* = 11 (92%)
Manual	*n* = 3 (23%)
Digital	*n* = 8 (69%)
**No**	*n* = 2 (15%)
Technology desired	*n* = 2 (15%)
**4. What other information, if any, do you document about your residents?**	
Yes, other information is tracked	*n* = 2 (15%)
Infractions	*n* = 1 (8%)
No other information tracked	*n* = 11 (85%)

## APPENDIX 2

Table A2

**Table A2:** House level data collection (*N* = 13), United States, 2020.

Question	Recovery house responses
**What information do you collect/document to support the business operations of your recovery house?**	
General financial information	*n* = 11 (85%)
Rent	*n* = 10 (77%)
**Does your house report data to an external organization? If so, to whom, and for what purpose?**	
Yes	*n* = 8 (62%)
Public Funder (County, State, Federal Grants)	*n* = 7 (54%)
No	*n* = 5 (38%)
**What types of reports would be valuable to you, your residents, or your business?**	
Business reports	*n* = 4 (31%)
Resident reports	*n* = 11 (85%)
**How do you track room and bed availability in your house(s)? Do you use software or some other system to indicate when a bed is open? Please describe this process**.	
Digital	*n* = 6 (46%)
Manual	*n* = 7 (54%)
